# Management of a purulent-necrotic surgical site infection following instrumented scoliosis correction: a case report

**DOI:** 10.3389/fsurg.2026.1859490

**Published:** 2026-10-26

**Authors:** Madina Rashova, Elmir Jamaleddinov, Konstantin Vasilyev, Berik Tuleubayev, Amina Koshanova, Zufar Bilalov

**Affiliations:** 1 Department of Traumatology and Neurosurgery, Karaganda Medical University, Karaganda, Kazakhstan; 2 Department of Molecular Biology, Biochemistry and Microbiology, Karaganda Medical University, Karaganda, Kazakhstan

**Keywords:** *Klebsiella pneumoniae*, purulent–necrotic wound infection, surgical site infection, thoracolumbar scoliosis, vacuum-assisted closure (VAC) therapy

## Abstract

Purulent–necrotic infections of the surgical wound following instrumented correction of thoracolumbar scoliosis remain among the most serious complications in spinal surgery, because they often require implant removal, prolonged disability, and recurrence. In adult patients undergoing surgery at a mature age for scoliosis deformity (traditionally corrected in childhood or adolescence), such complications occur less frequently and have specific clinical features. The presented clinical case of a 46-year-old patient with a postoperative wound infection in the form of purulent–necrotic phlegmon after correction of the thoracic spine illustrates successful preservation of spinal instrumentation after six revision and debridement procedures, followed by installation of a vacuum-assisted closure (VAC) system and two VAC system replacements with complete wound closure. This case suggests the potential value of repeated surgical debridement combined with vacuum-assisted therapy as an effective approach to implant salvage even in severe purulent-necrotic processes, which is of significant importance for clinical practice.

## 1 Introduction

Purulent-inflammatory complications following instrumental spinal surgery remain one of the leading causes of unfavorable outcomes in modern spinal surgery (1). The incidence of deep surgical site infections (SSI) after posterior instrumentation of the thoracic and lumbar spine ranges from 1%–2% in standard procedures to 5%–10% or higher in deformity correction, particularly in patients with additional risk factors (1, 2).

The clinical importance of managing these infections stems from their increasing incidence and the emergence of antibiotic-resistant strains (3). All risk factors for surgical wound suppuration can be divided into three groups: microbiological, surgical, and those related to the patient's medical condition (4).

Adult spinal deformity surgery is associated with substantially higher perioperative morbidity than corrective procedures performed in adolescents because of advanced age, multiple comorbidities, longer operative times, greater blood loss, and the need for extensive multilevel instrumentation. Consequently, deep surgical site infections remain among the most challenging postoperative complications in adult spinal surgery. Their management is particularly difficult because bacterial biofilms developing on implanted hardware markedly reduce antibiotic efficacy and often necessitate repeated surgical interventions or implant removal (14).

Although numerous studies have evaluated the management of postoperative spinal infections, reports describing successful preservation of long-segment instrumentation in adult patients with severe purulent-necrotic wound infection caused by extensively drug-resistant (XDR) *Klebsiella pneumoniae* remain limited. The present case demonstrates successful implant retention after six sequential surgical debridement procedures followed by staged vacuum-assisted closure (VAC) therapy and targeted antimicrobial treatment, resulting in complete wound healing without recurrence within six months. Thus, this report provides additional data confirming the strategy of implant preservation in a patient with severe postoperative spinal infection.

## 2 Clinical case

According to the patient, she had suffered from this condition since childhood and had been diagnosed with Grade IV (Cobb's angle is over 40) idiopathic S-shaped thoracolumbar scoliosis, which had been managed conservatively. She was followed by a traumatologist and neurologist at the place of residence (Figure 1).

**Figure 1:**
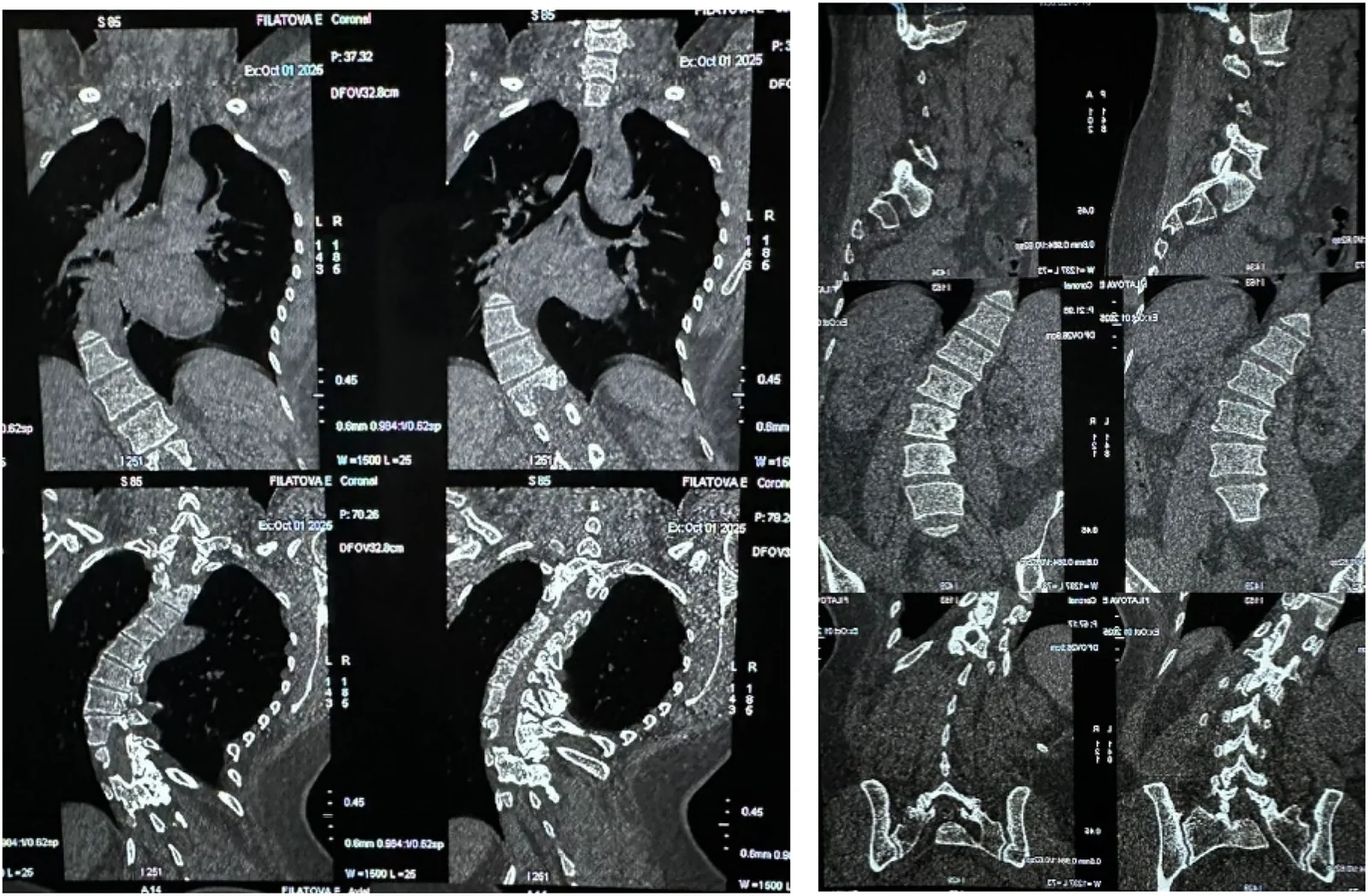
A CT scan before surgical treatment. Idiopathic scoliosis of the thoracolumbar spine grade IV.

She was consulted at the Research Institute of Traumatology and Orthopedics in Astana in 2020, where surgical treatment was recommended.

Five weeks prior to admission, the patient underwent surgery in Turkey for idiopathic S-shaped scoliosis (Figure 2). According to the available medical records from the treating institution, the patient underwent posterior segmental spinal instrumentation for scoliosis correction on 5 November 2025. However, detailed operative information, including the instrumented vertebral levels, implant system, operative time, estimated blood loss, and perioperative antibiotic prophylaxis, was not available in the documentation provided to our institution. According to the available radiographs, the patient underwent long-segment posterior pedicle screw instrumentation extending approximately from T2 to L3.

**Figure 2:**
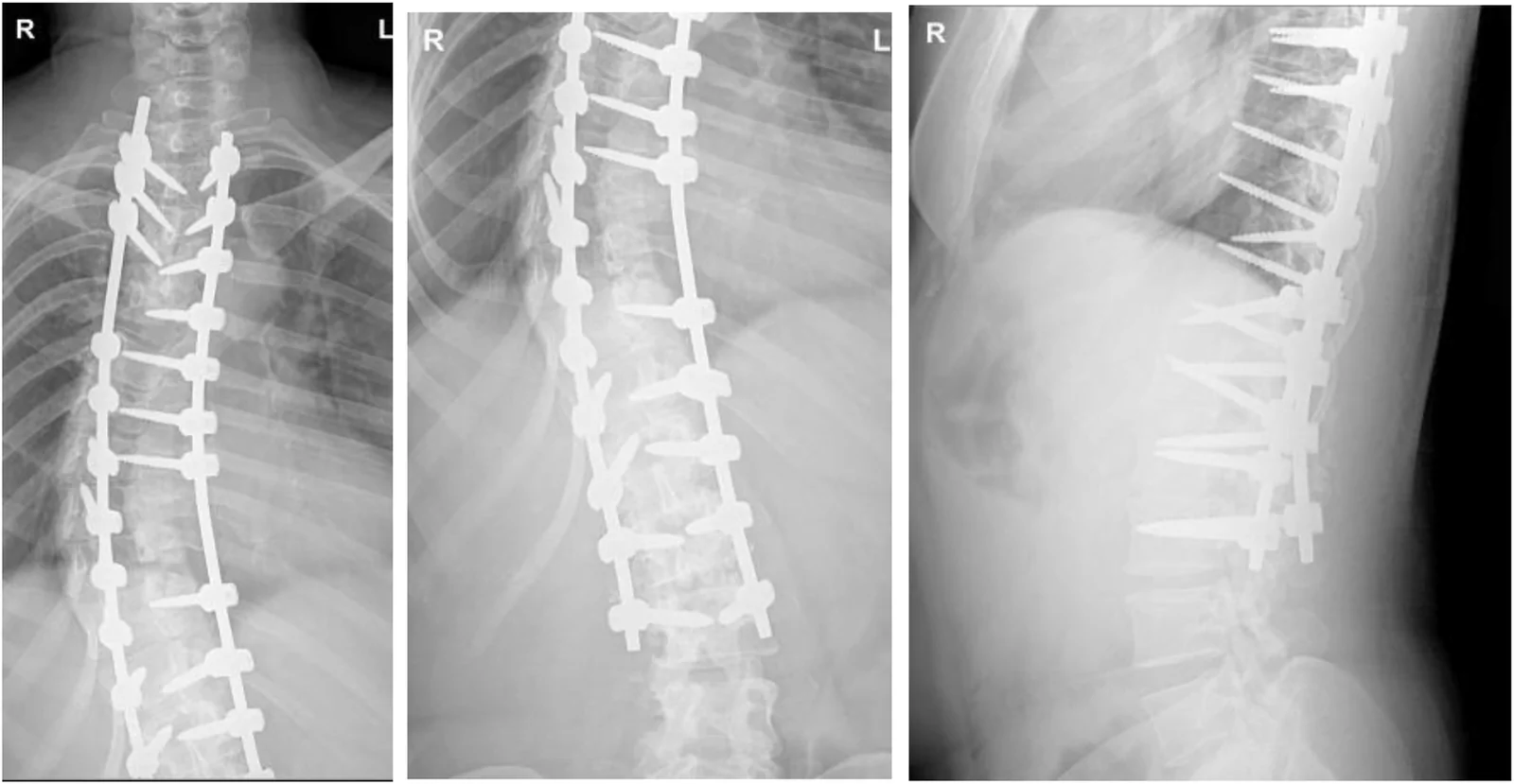
Multilevel transpedicular fixation and correction of scoliosis of the thoracolumbar spine with TAISI 5.11.25.

The patient presented to the emergency department of Professor Kh. Zh. Makazhanov Multidisciplinary Hospital in Karaganda, where after examination she was hospitalized in the combustiology department for surgical treatment (Figure 3).

**Figure 3:**
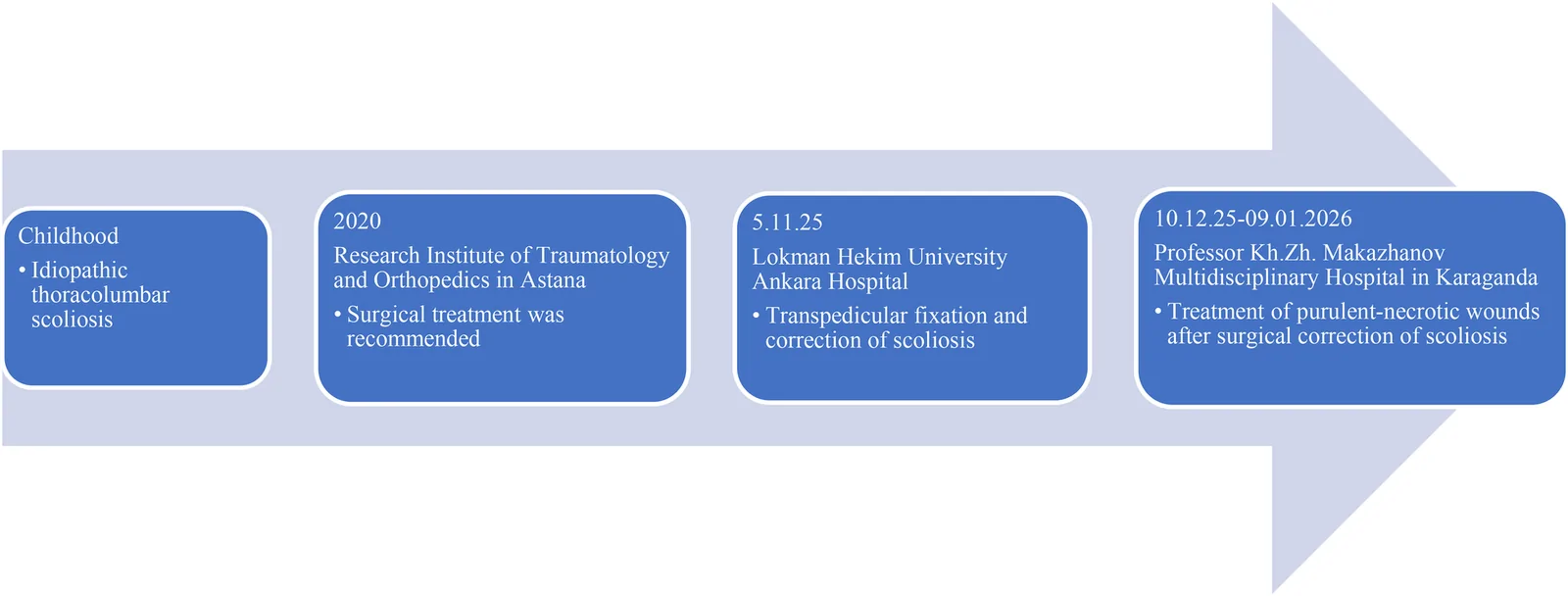
Chronological diagram.

Written informed consent for publication was obtained from the patient.

### 2.1 General examination

The patient's general condition is of moderate severity, mainly due to pain syndrome. Consciousness is clear. The patient assumes a passive position. Body temperature is 36.5 °C. Respiratory rate is 19 breaths per minute. Both halves of the chest participate equally in respiration. The chest was normally shaped. Oxygen saturation is 97%–98%. The heart area and peripheral vessels were unremarkable on visual examination. The apical impulse is localized. The borders of relative cardiac dullness are within normal limits. Heart rate is 78 beats per minute. Pulse is 78 beats per minute, rhythmic, with satisfactory filling, and symmetrical in both arms. Heart sounds are clear and rhythmic. Blood pressure is 140/90 mmHg.

### 2.2 Local examination

In the thoracic region of the back, separation of the edges of the postoperative wound is observed. Palpation is painful. The wound bed consists of poorly vascularized granulation tissue, with edema of the surrounding soft tissues and pain on movement (Figure 4). No neurogenic dysfunction is observed.

**Figure 4:**
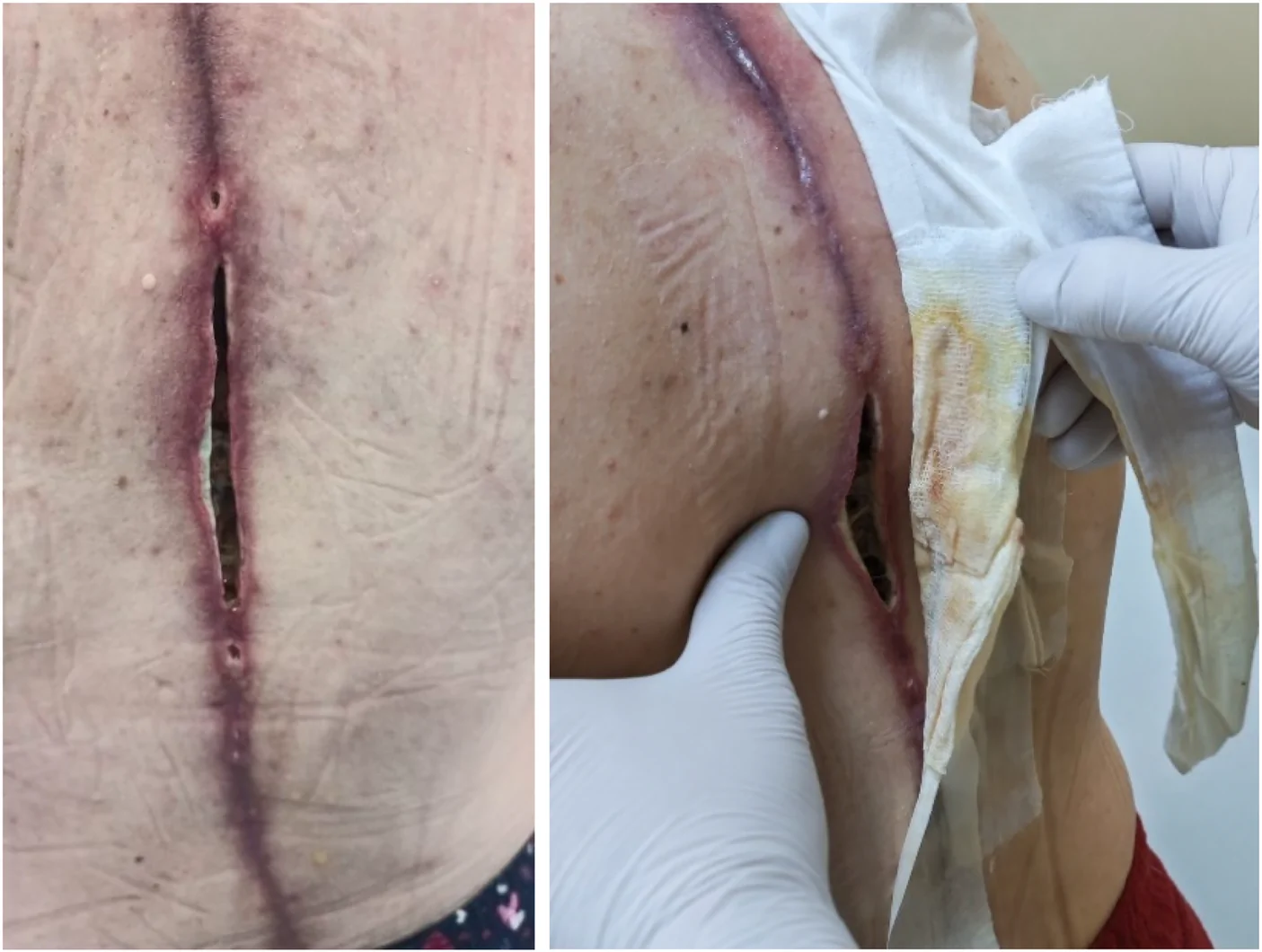
Condition of the postoperative wound at admission.

### 2.3 Laboratory findings

#### 2.3.1 Complete blood count (10.12.2025)

Erythrocyte sedimentation rate (ESR) measured manually – 11 mm/h; Leukocytes – 15.40 × 10⁹/L; Erythrocytes (RBC) – 3.49 × 10^12^/L; Hemoglobin (HGB) – 103 g/L; Hematocrit (HCT) – 31.0%; Platelets (PLT) – 318 × 10⁹/L; Lymphocytes (LYM%) – 4.5%; Absolute lymphocyte count (LYM) – 0.7 × 10⁹/L; Neutrophils (NEUT%) – 86.0%; Absolute neutrophil count (NEUT) – 13.2 × 10⁹/L; Mixed cells (monocytes, basophils, eosinophils) (MXD%) – 9.5%.

#### 2.3.2 Urinalysis (10.12.2025)

Urine protein – 0.00 g/L; Ketone bodies – negative; Leukocytes – 4/µL; Erythrocytes – 6/µL; Glucose – negative; Urine pH – 6.0; Bilirubin – negative; Urine clarity – turbid; Specific gravity – 1020; Urobilinogen – 3.5 µmol/L; Nitrites – negative; Ascorbic acid – negative; Urine color – yellow; Epithelial cells – 6/mL.

#### 2.3.3 Biochemical blood test (10.12.2025)

Total protein – 64.92 g/L; Alanine aminotransferase (ALT) – 37.8 U/L; Direct bilirubin – 3.30 µmol/L; Total bilirubin – 12.90 µmol/L; Aspartate aminotransferase (AST) – 19.7 U/L; Glucose – 6.9 mmol/L; Urea – 6.21 mmol/L; Creatinine – 63.65 µmol/L; C-reactive protein – 242.69 mg/L.

#### 2.3.4 Coagulation profile

Activated partial thromboplastin time (APTT) – 27.80 s; Thrombin time – 15.00 s; Fibrinogen – 9.007 g/L; Prothrombin time (PT) – 12.7 s; Prothrombin index (PTI) – 86.6%; International normalized ratio (INR) – 1.15.

#### 2.3.5 Microbiological cultures (simple swab)

Heavy growth of *Klebsiella pneumoniae* was noted from 15.12.25, 17.12.25 and 20.12.25; from 26.12.25, 31.12.25 and 06.01.26 – no bacterial growth.

Antimicrobial susceptibility testing revealed of *Klebsiella pneumoniae*: Amikacin – R; Ampicilin 10 µg + sulbactam 10 µg – R; Aztreonam – R; Cefotaxime – R; Ceftazidime – R; Ceftriaxone – R; Ciprofloxacin – R; Gentamicin – R; Imipenem – R; Meropenem – R; Moxifloxacin – R; Piperacillin+Tazobactam – R; Tigecycline – S.

### 2.4 Instrumental examinations

#### 2.4.1 Electrocardiography (12-lead ECG) (10.12.2025)

Sinus rhythm, HR 99 bpm, normal electrical axis, sinus tachycardia, incomplete right bundle branch block.

#### 2.4.2 Computed tomography of the chest and mediastinum (10.12.2025)

CT findings of peribronchovascular thickening of the pulmonary interstitium consistent with chronic bronchitis. Thoracolumbar spine deformity with grade IV scoliosis.

#### 2.4.3 Computed tomography of the abdominal cavity and retroperitoneal space (10.12.2025)

CT findings of hepatomegaly and biliary sludge in the gallbladder (cholelithiasis cannot be excluded).

#### 2.4.4 Neurosurgical consultation (10.12.2025)

Recommended: MRI of the thoracic spine (1.5 T) and MRI of the lumbar spine (1.5 T).

#### 2.4.5 Main diagnosis

Purulent-necrotic wound in the area of the postoperative wound of the thoracic spine. Condition after spinal correction surgery (5 November 2025).

### 2.5 Treatment

#### 2.5.1 Medical therapy

Sterofundin ISO 1,000 mL; Enoxaparin 4,000 anti-Xa IU/0.4 mL (40 mg); Omeprazole 20 mg; Ketoprofen 100 mg/2 mL; Diphenhydramine 1% (10 mg/mL); Cefotaxime sodium 1 g; Levofloxacin 5 mg/mL; Albumin 20%, 100 mL; Ibuprofen 800 mg/8 mL; Meropenem 1,000 mg; Amikacin 500 mg; Ferrous sulfate320 mg.

According to the bacteriological culture obtained on 15 December 2025, *Klebsiella pneumoniae* resistant to the current antimicrobial therapy was isolated. Therefore, antimicrobial treatment was changed to intravenous tigecycline (loading dose 100 mg followed by 50 mg every 12 h for 14 days).

#### 2.5.2 Surgical procedures: surgical wound debridement and revision

11.12.2025; 12.12.2025; 15.12.25; 17.12.2025; 19.12.2025; 22.12.2025.

VAC system placement was carried out in a continuous mode of 100 mmHg: 25.12.2025.

VAC replacement: 29.12.2025; 31.12.2025; 05.01.2026.

##### 2.5.2.1 Surgical procedure (12.12.2025)

Under intravenous anesthesia, the patient was placed on an orthopedic table in the right lateral position. Intraoperative findings included a wound along the midline of the back was identified and drained with gauze pads soaked with wound exudate; these were removed. After fourfold preparation of the surgical field with povidone-iodine, the wound was thoroughly irrigated with antiseptic solutions.

During revision: marginal inflammation persisted; the wound surface was debrided of sub-fascial necrotic tissue; elements of the metal fixation system were adjacent to the wound; areas of secondary necrosis were identified and excised. The wound was irrigated again with antiseptic solutions and hemostasis was verified. The wound was loosely drained with gauze pads soaked in povidone-iodine and 3% hydrogen peroxide. An aseptic dressing was applied. Estimated intraoperative blood loss: 50 mL (Figure 5).

**Figure 5:**
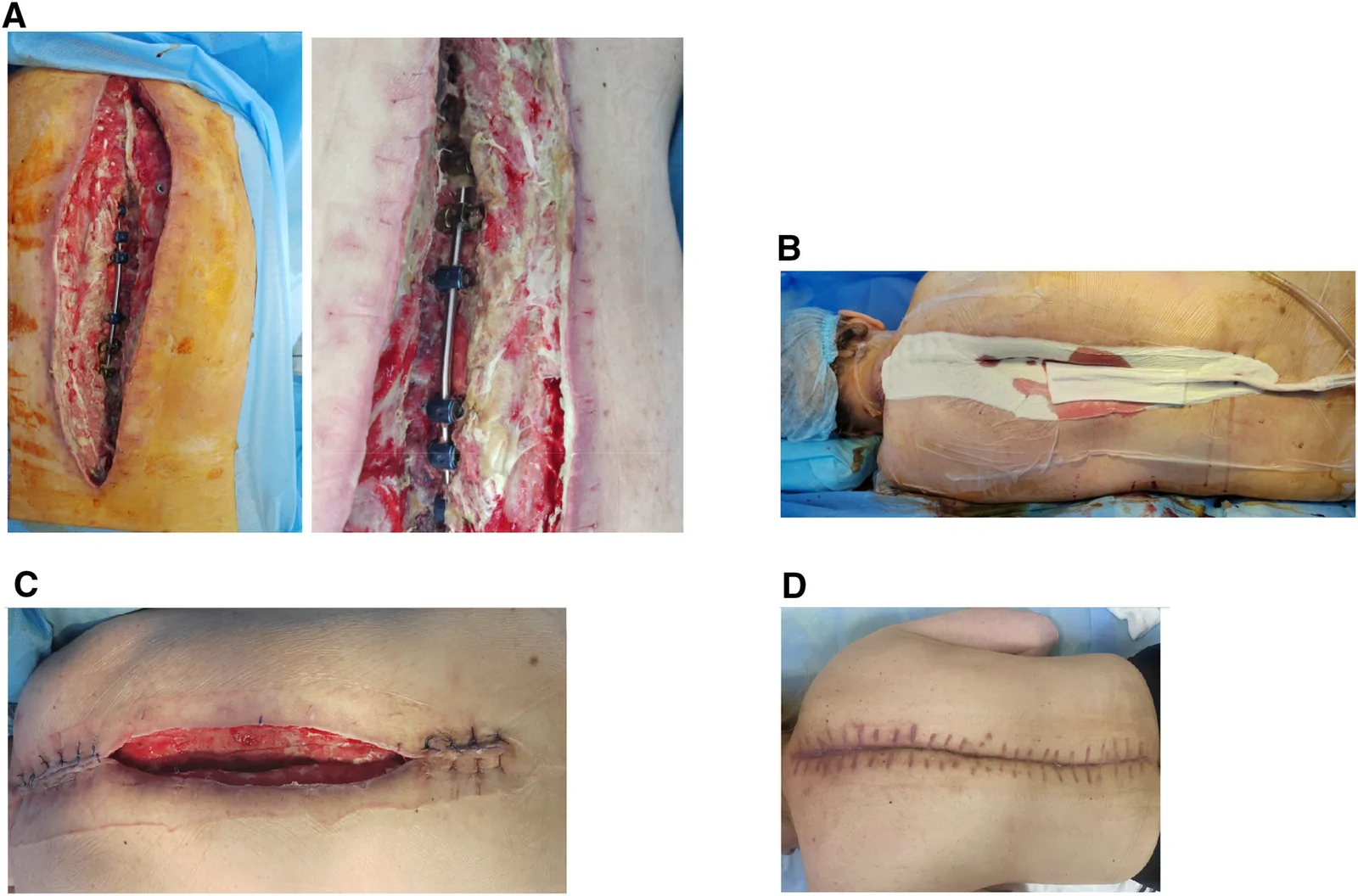
**(A–D)** stages of treatment. **(A)** 4 revision and debridement of wounds 17.12.2025. **(B)** The primary installation of the VAC 25.12.2025. **(C)** Partial suturing. Change of the VAC on 29.12.2025. **(D)** One month after the complete suturing of the wound 06.02.2026 on a repeat visit after discharge.

#### 2.5.3 Rehabilitation consultation

The patient’s condition was moderately severe due to pain syndrome.

Vital signs: BP 120/80 mmHg; HR 75 bpm; RR 18/min. Muscle strength according to the Lovett scale: Left lower extremity — 5.0 points; Right lower extremity — 5.0 points.

Rehabilitation program: Passive kinesiotherapy of the upper extremities with gradual patient mobilization under supervision of a physiotherapy instructor. Passive range-of-motion exercises for the upper extremities to improve muscle strength and tone; slow tempo, incomplete amplitude, minimal load.

Breathing exercises. Magnetic therapy to the thoracic spine.

## 3 Discussion

Various approaches to the treatment of such complications are described in the literature, ranging from early revision with removal of the spinal instrumentation to attempts to preserve implants using prolonged antibiotic therapy, repeated surgical debridement, and modern wound management techniques including vacuum-assisted closure therapy (VAC/NPWT) (5). Recent reviews and case series have demonstrated that with early diagnosis and adequate management, implant preservation can be achieved in 80%–92% of cases, significantly reducing the risk of loss of deformity correction, repeated major surgeries, and deterioration of neurological status (6).

Cases involving adult patients are of particular interest in adult patients with late-diagnosed and surgically corrected scoliosis, since most scoliosis corrections are performed during childhood or adolescence. Surgical procedures in adults are associated with greater technical complexity, poorer tissue vascularization, and an increased risk of infectious complications.

Purulent-necrotic phlegmon as a manifestation of deep postoperative wound infection following thoracic spinal fusion represents a severe condition, often requiring multi-stage surgical treatment and frequently resulting in implant removal.

In the presented clinical case, a 46-year-old woman developed a postoperative wound infection with the formation of purulent-necrotic phlegmon after correction of thoracolumbar scoliosis (November 2025).

The etiological factor in this clinical example was *Klebsiella pneumoniae*, a genus of opportunistic bacteria belonging to the family of Enterobacteria. *Klebsiella pneumoniae* is one of the most significant opportunistic pathogens causing both local purulent-inflammatory diseases and severe systemic infections infections, including severe, often life—threatening conditions—meningitis and sepsis (7). The main virulence factors of *Klebsiella pneumoniae* include a polysaccharide capsule that protects against phagocytosis, endotoxin (LPS), mannose-resistant pili for adhesion, as well as a hypermucoid phenotype and biofilm formation that provide resistance to immunity and antibiotics (7, 8). These mechanisms allow *Klebsiella* to cause severe nosocomial infections. Antibiotic-resistant representatives of this species are the main cause of mortality in hospital patients worldwide, and, along with other pathogens, are classified by WHO as critically important pathogens for the development of antibacterial drugs (9). In the present case, the isolated *Klebsiella pneumoniae* exhibited an extensively drug-resistant (XDR) phenotype and remained susceptible only to tigecycline. Such resistance patterns substantially complicate the management of deep spinal implant-associated infections because of limited therapeutic options and the ability of the pathogen to form biofilms on implant surfaces. Therefore, successful management requires both effective surgical source control and targeted antimicrobial therapy.

Negative pressure wound therapy (NPWT) was intentionally delayed after the initial intervention, since in the early postoperative period there were pronounced signs of a purulent infection with the presence of necrotic tissues and the need for staged surgical treatments. At this stage, the priority was adequate drainage of the wound, dynamic control of tissue viability and consistent debridement of the infection site. After performing a series of surgical procedures, achieving adequate control of the infectious process, removing non-viable tissues and forming a wound bed suitable for the use of negative pressure, a vacuum-assisted closure (VAC) system was installed on 25.12.2025. Thus, the delayed use of NPWT corresponded to the principles of step-by-step treatment of infected wounds and was conditioned by the need for preliminary radical sanitation of the purulent focus.

Despite the severity of the infection, it was possible to preserve the spinal instrumentation through six surgical revisions and wound debridement procedures, followed by VAC therapy with two replacements and complete secondary wound closure without recurrence within six months. Compared with conventional dressings, VAC therapy offers several advantages. The polyurethane foam dressing facilitates continuous removal of wound exudate. An occlusive adhesive film seals the wound and minimizes external contamination. Negative pressure reduces tissue edema, improves local perfusion, and promotes granulation tissue formation. In addition, gradual approximation of the wound edges facilitates secondary wound closure. An occlusive adhesive film covering the dressing insulates and seals the entire wound area, preventing bacterial contamination from both the external environment and the wound itself. The principles of the VAC system: active cleansing due to the continuous removal of wound exudate, pus and bacteria from the wound; stimulation of blood circulation by creating negative pressure; reduction of edema due to the removal of excess fluid from the intercellular space, which reduces pressure on the tissue and improves lymph flow. Stimulation of granulation: constant vacuum exposure promotes the active growth of new, healthy granulation tissue, as well as the tightening of wound edges: the physical effect of vacuum brings the edges of the wound defect closer, reducing its area and creating a sealed environment (10–12). Previous studies have similarly emphasized that successful treatment of purulent-necrotic infections relies on adequate surgical debridement, effective local wound management, and consideration of antimicrobial resistance and biofilm formation (13, 14).

A limitation of this case is that susceptibility testing for colistin and newer anti-carbapenem-resistant agents (e.g., ceftazidime-avibactam or cefiderocol) was not available. Consequently, antimicrobial treatment was selected according to the local susceptibility profile, in which tigecycline was the only active agent identified. In our clinical practice, successful preservation of thoracic spinal instrumentation in patients with such severe postoperative infections is uncommon, making this case valuable from both educational and clinical perspectives. Further multicenter studies are needed to determine whether similar outcomes can be achieved in larger patient populations.

## 4 Conclusion

This clinical case demonstrates that successful preservation of spinal instrumentation may be achievable in selected patients with severe postoperative purulent-necrotic wound infection through repeated surgical debridement, targeted antimicrobial therapy, and staged vacuum-assisted closure (VAC) therapy. Although favorable clinical outcomes were observed in this patient, conclusions regarding the effectiveness of this treatment strategy cannot be generalized from a single case. Further prospective studies and larger case series are needed to evaluate the safety, reproducibility, and long-term outcomes of implant-preserving approaches in patients with deep postoperative spinal infections caused by multidrug-resistant pathogens.

## Data Availability

The original contributions presented in the study are included in the article/Supplementary Material, further inquiries can be directed to the corresponding author.
